# Inhibition of STAT3 signaling induces apoptosis and suppresses growth of lung cancer: good and bad

**DOI:** 10.1186/s42826-019-0030-0

**Published:** 2019-12-21

**Authors:** Ju-Hee Kang, Yeong-Su Jang, Ha Jung Lee, Chang-Yong Lee, Dong Yun Shin, Seung Hyun Oh

**Affiliations:** College of Pharmacy, Gachon University, Incheon, Republic of Korea

**Keywords:** STX-0119, STAT3 inhibitor, NSCLC, Xenograft, Cancer

## Abstract

Signal transducer and activator of transcription 3 (STAT3) modulates a variety of genes involved in the regulation of critical functions, including cell proliferation, differentiation, apoptosis, angiogenesis, metastasis, and immunity. For many cancers, elevated levels of STAT3 signaling have been associated with a poor prognosis and the development of chemotherapy resistance. In this study, we investigated the inhibitory effects of a novel small-molecule inhibitor of STAT3, STX-0119, on the cell viability and survival of human lung cancer cells. STX-0119 inhibited activated STAT3 and the expression of STAT3-regulated oncoproteins such as c-Myc, cyclin D1, and survivin in lung cancer cells. STX-0119 also decreased the amount of STAT3 in the nuclear fraction as well as induced apoptosis of these lung cancer cell lines as evidenced by increases in apoptotic cells (Annexin V positive) and poly (ADP-ribose) polymerase (PARP) cleavage. The efficacy of STX-0119 in a mouse xenograft model was confirmed. However, a hematological side effect, which had not been previously reported, was observed. The level of white blood cells was significantly lowered when treated at the dose at which STX-0119 alone showed a significant tumor-suppressive effect. In conclusion, we suggest that STX-0119 may be a potent therapeutic agent against lung cancer. Consideration of the side effect suggests, it is necessary to study whether low-dose STX-0119 is effective for lung treatment with a combination of classic lung cancer therapeutics.

## Introduction

The signal transducer and activator of transcription (STAT) family of proteins is a group of transcription factors that regulate gene expression related to the cell cycle, cell survival, and the immune response. In normal cells, STAT3, originally identified as a mediator of the acute phase of the inflammatory response triggered by interleukin-6, is under tight control. STAT3 is particularly interesting because of its constitutive activation found in various types of solid tumors and hematological tumors [1]. Although a few researchers have suggested a tumor-suppressive role of STAT3, the predominant view is that inappropriate STAT3 activation contributes to tumor development and progression through various cytokines and growth factors [2, 3].

STAT3 has been reported to be involved in oncogenesis by up-regulating the transcription of several genes that control primary tumor cell survival, resistance to apoptosis, cell cycle activation, and angiogenesis [4–7] . Targets of STAT3 include anti-apoptotic (Bcl-2, Bcl-xL), proliferative (cyclin D1), and angiogenic (VEGF) proteins [8]. Dysregulated STAT3 activity is involved in hematologic malignancies, head and neck cancers, and leukemia and has been implicated in the pathogenesis of a subset of solid tumors [9–11]. In addition, there have been reports that STAT3 activation maintains cancer stem cell phenotypes, facilitating drug resistance and tumor recurrence [12].

Since its pivotal roles in inflammation-related diseases and neoplasm have been highlighted, the rationale for developing small molecules targeting the STAT3 signaling pathway is solid. A number of IL-6 blocking antibodies and IL-6 receptor blocking antibodies, STAT3 inhibitors, and JAK inhibitors have been reported [1]. Among them, tocilizumab, an IL-6 receptor inhibitor, and ruxolitinib, a JAK1/2 and tyrosine kinase 2 inhibitor, have been approved for use against rheumatoid arthritis and myeloproliferative neoplasms, respectively [13]. The mechanisms of action of direct STAT3 inhibitors vary considerably. They include disrupting phosphorylation (Stattic and OPB-51602), dimerization (STX-0119 and K116), transcriptional activity (ST3-Hel2A-2), and/or DNA-binding of STAT3 (CPAs and c48) and various classes of STAT3-inhibiting small molecules have been developed [1, 14–17]. Nevertheless, there are no clinically approved drugs directly targeting STAT3. There are some remaining problems related to direct STAT3 inhibition. Unlike the inhibitors of enzymes or cell surface receptors, both of which amplify cellular signaling, STAT3 molecules are able to transduce without amplification. Therefore, complete inhibition of STAT3 signaling pathway requires a high drug concentration [18]. Also, STAT3 is ubiquitously expressed throughout the entire body and regulates a wide range of genes related to the cell cycle, cell survival, and the immune responses, and, as a direct inhibitor, easily producing off-target toxicities [14, 15, 19].

Lung cancer is the leading cause of cancer death in men and women, both worldwide and in less developed countries, resulting in 1.18 million deaths each year [20]. Non-small cell lung cancer (NSCLC) accounts for 80 to 85% of lung cancers. Only 18.1% of all lung cancer patients are alive at 5 years post-diagnosis or more [21]. There is a wide variety of chemotherapy options for NSCLC, but difficulties remain in significantly improving survival in advanced NSCLC [22]. Furthermore, early NSCLC is usually not sensitive to chemotherapy and/or radiation, as a result, surgery remains to be the main treatment of choice. Thus, finding an effective chemotherapeutic agent to treat lung cancer is urgently needed. Recently, poor prognoses in cancer patients with high STAT3 expression have been reported [23–25]. In 22%~ 65% of NSCLC, STAT3 is reported to be over-activated, and high levels of STAT3 or phospho-STAT3 expression are a strong predictor of a poor prognosis for patients with NSCLC [14, 26].

A novel STAT3 inhibitor, STX-0119 has been identified and showed anti-cancer effect with low toxicity [16, 27–29]. However, it was not tested in lung cancer which is major type in worldwide. In the present study, we investigated the effect of STX-0119, which inhibiting STAT3 dimerization, on the cell growth in lung cancer cell lines in vitro and in vivo. Considering the importance of STAT3 in hematopoiesis and innate immunity, we also examined the side effect of STX-0119 by blood cell differential counts. The results demonstrate that the STX-0119 molecule can significantly inhibit the viability and survival of lung cancer cell lines and can decrease tumor size in mice bearing transplanted A549 lung cancer cells.

## Materials and methods

### Cell lines and reagents

The lung cancer cell lines H1299, A549, and H23 were purchased from the American Type Culture Collection (Manassas, VA, USA). All cell lines were cultured in RPMI1640 medium, 100 U/mL penicillin, 100 μg/mL streptomycin, and 10% (v/v) fetal bovine serum (WelGENE, Daegu, Korea). Various antibodies against STAT3, phospho-specific STAT3 (Tyr 705), c-Myc, cyclin D1, survivin, PARP, cleaved PARP, α-tubulin, and fibrillarin were purchased from Cell Signaling Technology, Inc. (Danvers, MA, USA) and EMD Millipore (Billerica, MA, USA) for use in western blotting. The ApopTag® Red In Situ Apoptosis Detection Kit (TUNEL) was purchased from EMD Millipore (Billerica, MA, USA). LPS purified from *Escherichia coli* O111:B4 was purchased from Sigma-Aldrich (St. Louis, MO, USA). The STX-0119 (Stat3 inhibitor) was synthesized by Dr. Dong Yun Shin, Gachon University, Republic of Korea.

### Cell viability assay

Cell viability was determined by using the MTT assay (Biosesang, Seongnam, Korea). Cells (*n* = 3000) were seeded into each well of a 96-well plate (SPL Life Sciences, Seoul, Korea) and treated with various doses of STX-0119. After incubation for 48 h at 37 °C, 10 μL of MTT solution was added to each well followed by incubation for 2 h at 37 °C. The absorbance of each well was measured in a microtiter plate reader at 570 nm (Biotek, Winooski, VT, USA).

### Colony formation assay

For the colony formation assay, H1299, A549, and H23 cells were cultured in RPMI medium. Cells were then plated at a density of 500 cells per well in six-well plates. The lung cancer cells were treated with STA-0119 at various concentrations for 48 h. After 2 weeks incubation, cells were washed with PBS, fixed in methanol, stained with hematoxylin, and the colonies were counted. We then counted the number of colonies measuring ≥30 μm were counted.

### Apoptosis assay

The Annexin V/PI apoptosis kit from Santa Cruz was used for the assay (Santa Cruz, CA, USA). After cells were washed twice with cold PBS, they were resuspended in 1 × assay buffer at a concentration of 2 × 10^5^ cells/mL, after which 100 μL was transferred to a 5 mL tube containing 0.2 μg of Annexin-V-FITC and 10 μL of PI and incubated for 15 min. Four hundred microliters of 1 × assay buffer were added to the samples before the mixture was analyzed by using a BD FACS Calibur cytometer (BD Biosciences, CA, USA).

### Nuclear and cytoplasmic fractionation of lung cancer cells

The nuclear and cytoplasmic fractions from A549 cells were prepared by using a nonionic detergent method. In brief, nuclear extracts were prepared by exposure to extraction buffer (10 mM HEPES, pH 7.9, 10 mM KCl, 0.1 mM EDTA, 1 mM dithiothreitol) and protease inhibitors. After centrifugation at 14,000 rpm for 3 min, the supernatant fraction (cytoplasmic extract) was placed in new tubes, and the pellet was suspended in extraction buffer (20 mM HEPES, pH 7.9, 400 mM NaCl, 1 mM EDTA, 10% glycerol, 1 mM dithiothreitol, and 20% NP-40) with protease inhibitors. Then, after centrifugation at 14,000 r/min for 5 min, the supernatant fraction was the nuclear extract.

### Western blotting

Equal amounts of protein were separated on 8–12% SDS-PAGE gels and wet transferred to polyvinylidene difluoride membranes. The membranes were blocked with 5% skim milk, incubated with the respective antibodies overnight at 4 °C, and incubated with HRP-conjugated secondary antibody for 1 h. Finally, the immunoreactive bands were detected by Absignal (Abclone, Seoul, Korea).

### Animal experiments

All animal procedures were conducted in accordance with a protocol approved by the Institutional Animal Care and Usage Committee at Gachon University in Incheon, Korea [GIACUC-R2016004–1]. To determine the antitumor effect of STX-0119 in animals, we used a xenograft tumor model. For that purpose, A549 cells (5 × 10^6^ cells) were subcutaneously injected into the flank region of athymic nude mice. Two weeks after cancer cell injection, STX-0119 (80 mg/kg or 160 mg/kg, three times a week) was administered orally for 3 weeks. Tumor volume was measured by using calipers and was calculated according to the formula (length × width^2^)/2. All mice were sacrificed on day 35 post-injection, and tumor tissues were isolated from them. The results are presented as mean and SEM tumor volumes (*n* = 7 per group).

### Histological analysis

The formalin-fixed tumor tissues were processed, embedded in paraffin, and underwent hematoxylin and eosin staining. Immunohistochemical analysis performed as described previously [30]. Tumor tissues sections were reacted with appropriately diluted primary antibodies for cyclin D1 (1:50) and c-Myc (1:50) and incubated with biotinylated goat anti-rabbit secondary antibody and avidin-biotin-peroxidase. Sections were visualized by chromogen 3,3′-diaminobenzidine (Dako, K3466, USA) and counterstained with hematoxylin. TUNEL staining was performed in vivo by using the ApopTag Red In Situ Apoptosis Detection Kit (EMD Millipore) according to the manufacturer’s instructions. The intensity and localization of the immunoreactivity against antibodies were viewed under the light microscope (× 200).

### Statistical analysis

Values are presented as means ± standard deviations (SD) of three independent experiments. Differences between groups were examined by performing two-sample paired Student’s t-tests and a *p* value < 0.05 was considered to indicate a statistically significant difference between values.

## Results

### Effect of STX-0119 on STAT3 phosphorylation and STAT3 target-gene expressions

**Fig. 1:**
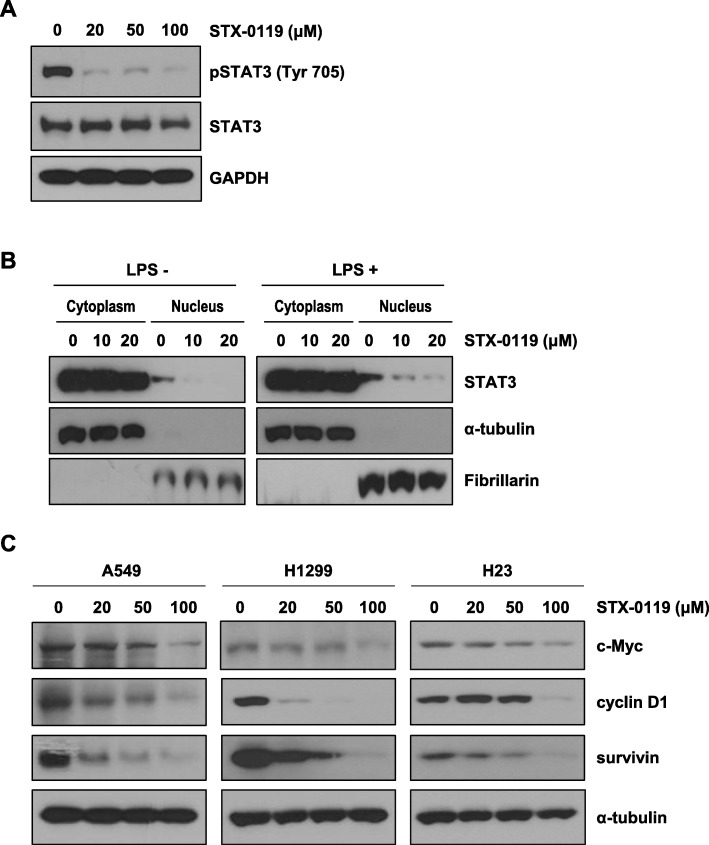
Suppression of STAT3 target-gene expressions by STX-0119 in lung cancer cells. **a** Effect of STX-0119 on Tyr705 phosphorylation of STAT3 in A549 cells. Cells were treated with indicated doses of STX-0119 for 48 h. **b** Subcellular localization of STAT3 in STX-0119-treated A549 cells. **c** Protein expressions of c-Myc, cyclin D1, and survivin in A549 cells after STX-0119 treatment for 48 h

To examine the ability of STX-0119 to inhibit STAT3 phosphorylation and dimerization in cells, western blotting analysis was performed. As shown in Fig. 1a, STAT3 phosphorylation was suppressed in A549 cells by STX-0119 treatment. To determine whether STAT3 localization was reduced by STX-0119, protein subcellular fractionation was performed. STAT3 is located mainly in the cytosol and nuclear fractions, and STX-0119 treatment decreased the amount of STAT3 in the nuclear, but not in the cytosol fraction (Fig. 1b). In addition, western blotting analysis of lysate from lung cancer cells treated with STX-0119 showed that STX-0119 decreased the expression of STAT3 target proteins such as c-Myc, cyclin D1, and survivin in a concentration-dependent manner (Fig. 1c).

### STX-0119 inhibits viability and clonogenic ability of lung cancer cells

**Fig. 2:**
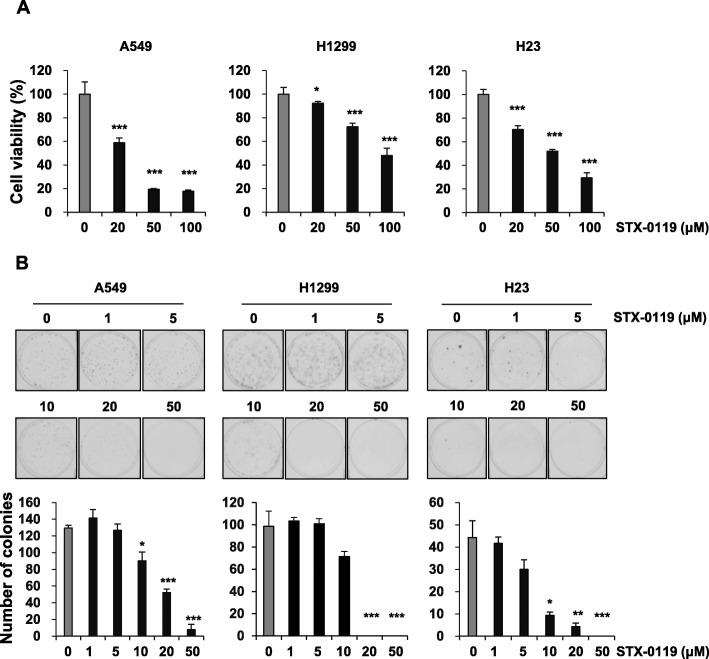
Inhibitory effect of STX-0119 on viability and survival of lung cancer cells. **a** Cell proliferation was measured after A549, H1299 and H23 cells were treated with the indicated concentrations of STX-0119 for 48 h. **b** Anchorage-dependent colony formation assays for cancer cells were performed following treatment with STX-0119 for 2 weeks. Representative pictures of colonies (upper panel) and quantitative analysis of colony numbers (bottom panel). Values presented are means ± SD. Statistical analysis was performed by applying Student’s *t*-test (*p < 0.05, **p < 0.005)

To test whether STX-0119 affects cell viability, we measured the proliferation of lung cancer cells after STX-0119 treatment by performing MTT assays. Exposure of the lung cancer cells to STX-0119 caused a dose-dependent decrease in cell viability (Fig. 2a). To determine whether the survival ability of lung cancer cells was also suppressed by STX-0119, the number of colonies produced in clonogenic assays was counted. Shown in Fig. 2b (upper panel) is a representative picture of the colony formation following treatment with different doses of STX-0119. The number of colonies was significantly reduced in a dose-dependent manner after treatment with STX-0119 (Fig. 2b, bottom panel). The number of colonies of A549 cells was significantly decreased to 52.3 ± 4.2 in the 20 μM STX-0119-treated group from that in the control group (129.7 ± 3.1 colonies). The results also showed that STX-0119 inhibited colony formation in H1299 and H23 cells (Fig. 2b).

### Induction of apoptosis by STX-0119 in lung cancer cells

**Fig. 3:**
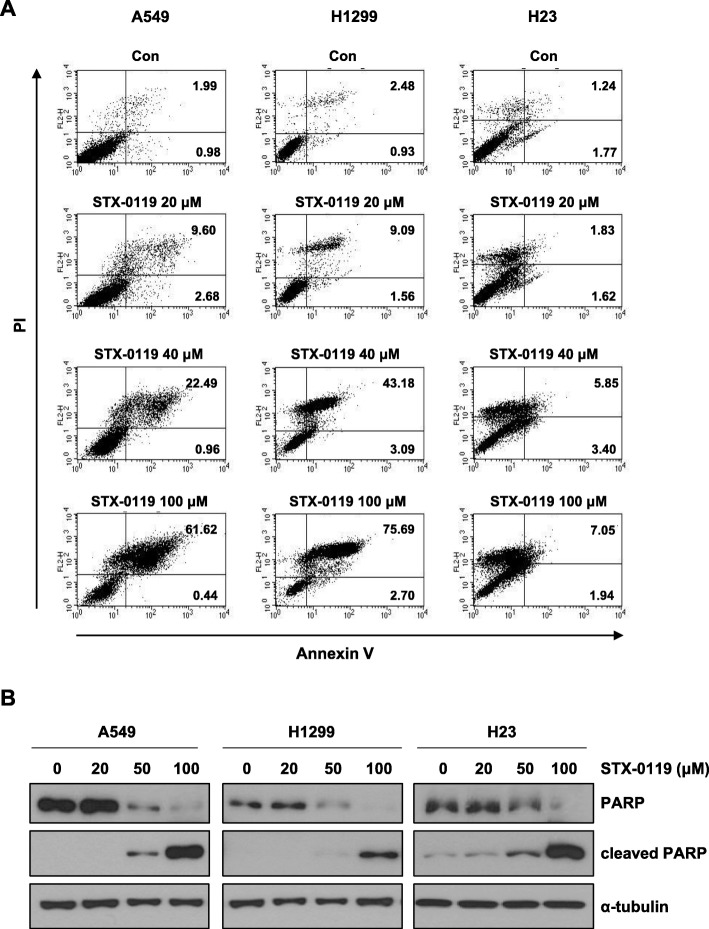
Induction of apoptosis in lung cancer cells by STX-0119. **a** After incubation with STX-0119 for 48 h, H1299, A549, and H23 cells were stained with Annexin V and PI solutions. **b** A549, H1299, and H23 cells were treated with STX-0119 for 48 h, followed by western blot analyses of whole-cell lysates using an anti-PARP antibody that recognizes cleaved PARP

To determine whether STX-0119 decreased cell proliferation by inducing apoptosis, lung cancer cells were cultured with STX-0119 at different concentrations (20, 40 or 100 μM) for 48 h and then assessed with Annexin V/PI staining assay. As shown in Fig. 3a, flow cytometry analysis revealed that the percentage of apoptotic cells with Annexin V/PI labeled cells increased gradually with STX-0119 concentration in STX-0119-treated cells. In A549 cells, the percentage of apoptotic cells increased from 3.0% in control cells to 12.3, 23.5, or 62.1% in cells treated with 20, 40, or 100 μM STX-0119, respectively. Similar results were obtained for H1299 and H23 cells when they were treated with the 20, 40, or 100 μM doses of STX-0119 (Fig. 3a). For a further assessment of apoptosis induced by STX-0119, we examined the levels of cleaved PARP proteins by performing western blotting in lung cancer cells. The results demonstrated that there was a significant dose-dependent increase in protein levels of cleaved PARP in each of the three lung cancer cell lines (Fig. 3b). Collectively, these data provided strong evidence that the STX-0119 can induce apoptosis in lung cancer cell lines, which may account for the observed reduction of cell viability and survival.

### STX-0119 suppresses tumor growth in a xenograft model of lung cancer

**Fig. 4:**
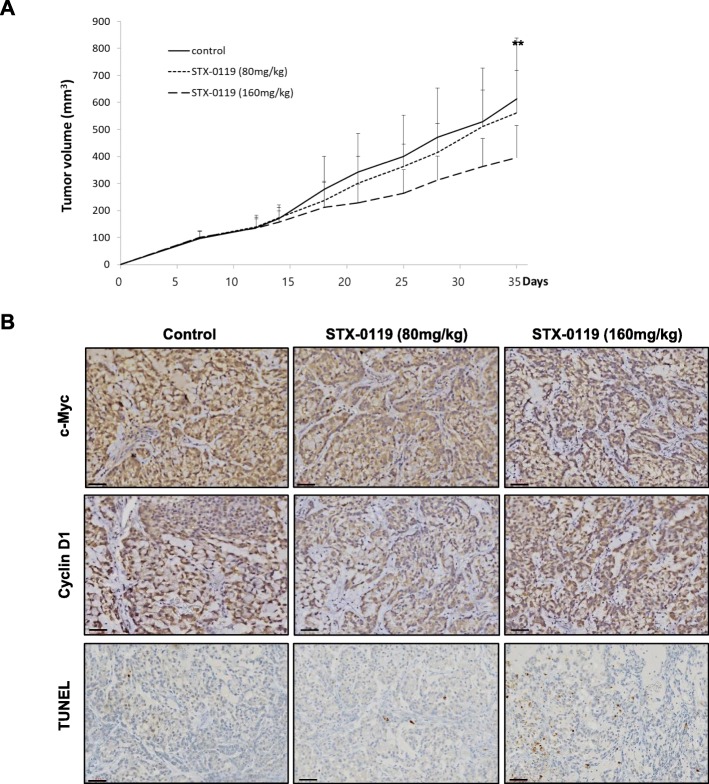
Inhibitory effect of STX-0119 on tumor growth of A549 cells. **a** Athymic nude mice were xenografted with lung cancer cells and administered STX-0119 orally three times a week for 3 weeks. **b** Immunohistochemistry results for c-Myc, cyclin D1, and TUNEL in mouse tumor tissue as visualized by light microscope (× 200). Scale bar, 60 μm

To further confirm the anti-cancer effect of STX-0119, we used an in vivo mouse model. We established an athymic nude mouse tumor xenograft model by inoculation of A549 cells. Tumor volume was measured twice a week for 5 weeks after the initial STX-0119 treatment. It showed that STX-0119 treated at 80 mg/kg did not have a significant inhibitory effect on tumor volume, but tumor volume decreased remarkably when treated at an STX-0119 dose of 160 mg/kg (Fig. 4a). Mean tumor volumes were as follows: vehicle control group, 613.6 ± 225.2 μm^3^; 80 mg/kg STX-0119-treated group, 560.7 ± 158.1 μm^3^; and 160 mg/kg STX-0119-treated group, 394.9 ± 119.2 μm^3^. The treatmetn of STX-0119 was also significantly correlated with the expression levels of c-Myc, cyclinD1, and TUNEL in tumor tissues. The IHC results showed that STX-0119 at the dose of 80 mg/kg and 160 mg/kg reduced the expression of c-Myc and cyclin D1 protein, while only 160 mg/kg STX-0119 increased the number of apoptotic cells in tumor tissues (Fig. 4b). These results demonstrate that STX-0119 inhibits the nuclear levels of STAT3, which ultimately results in reduced transcription of c-Myc and cyclin D1 (Fig. 4b). Collectively, these results demonstrate that STX-0119 suppresses tumor growth. No significant side effects were found in other organs except in bone marrow (Table 1). We conducted an analysis of the hematological or biochemical parameters of mice treated with STX-0119 for 3 weeks. The results showed no significant differences between parameters of the control and 80 mg/kg STX-0119-treated groups, but the white blood cell (WBC) numbers did decrease in the 160 mg/kg STX-0119-treated group, indicating that there would be an increased risk of immunosuppression at the higher dose level.

**Table 1:** Hematological analysis data of STX-0119 treated mice

Parameter	Control (*n*=8)	STX-0119 (80mg/kg, *n*=7)		STX-0119 (160mg/kg, *n*=7)	
	Mean±SD	Mean±SD	*p*-value(T-test)	Mean±SD	*p*-value(T-test)
WBC(X10³ cell/μl)	5.59±2.25	4.58±2.5	0.444	2.49±.87	0.005
RBC(X106 cell/μl)	8.29±.82	8.37±.60	0.839	8.32±.48	0.929
HGB(g/dl)	13.51±.69	13.17±.91	0.444	13.01±.68	0.199
HCT(%) (37-55)	45.00±3.40	44.83±3.48	0.927	42.94±1.53	0.170
MCV(fL)	54.47±2.57	53.6±1.89	0.484	51.7±1.34	0.026
MCH(pg)	16.41±1.17	15.77±.63	0.224	15.66±.57	0.149
MCHC(g/dl)	30.13±1.59	29.41±.48	0.277	30.33±1.05	0.786
RDW(%)	14.11±2.73	13.04±1.06	0.352	13.27±.95	0.456
PLT(X10³ cell/μl)	1014.86±239.79	1273.86±191.19	0.045	1116.29±201.41	0.408
MPV(fL)	5.79±0.34	5.94±0.28	0.368	6.19±0.72	0.207
NEUT(%)	26.27±6.18	30.96±6.87	0.205	29.26±5.23	0.349
LYM(%)	65.26±5.65	62.17±6.09	0.345	63.27±4.42	0.478
MONO(%)	0.8±0.24	1.03±0.43	0.243	0.8±0.38	1.000
EOS(%)	1.46±0.42	1.87±1.09	0.366	2.37±0.63	0.008
LUC(%)	6.11±5.58	3.86±2.68	0.354	4.16±5.77	0.531
BASO(%)	0.07±0.08	0.11±0.09	0.354	0.11±0.11	0.403

*WBC* White Blood Cells, *RBC* Red Blood Cells, *HGB* Hemoglobin, *HCT* Hematocrit, *MCV* Mean Corpuscular Volume, *MCH* Mean Corpuscular Hemoglobin, *MCHC* Mean Corpuscular Hemoglobin Concentration, *RDW* Red Cell Distribution Width, *PLT* Platelets, *MPV* Mean Platelet Volume, *NEU* Neutrophils (Sometimes labeled GR or Grans.), *LYM* Lymphocytes, *MONO* Monocytes, *EOS* Eosinophils, *LUC* Large unstained cell (peroxidase negative), *BASO* Basophils

## Discussion

STAT3 is a transcription factor that mediates expressions of genes known to be involved in several cellular processes such as proliferation, survival, and inflammation [4–6, 31]. Recent evidence from a number of studies has shown that STAT3 is abnormally upregulated in many solid and hematological tumors, providing prognostic information on such tumors [32]. Although there are many strategies for targeting the STAT3 signaling pathway, only indirect inhibitors, such as the JAK and tyrosine kinase inhibitors, have received FDA approval for use against rheumatoid arthritis and myeloproliferative neoplasm [15].

In this study, we investigated a novel small-molecule inhibitor, named STX-0119, to target STAT3 in lung cancer cells. STX-0119 inhibited persistent STAT3 phosphorylation and induced apoptosis in lung cancer cell lines. The inhibition of STAT3 signaling by STX-0119 was confirmed by down-regulating the expression of the downstream targets of STAT3, including c-Myc, cyclin D1, and survivin. In addition, STX-0119 decreased the LPS-induced nuclear localization of STAT3, but did not block the amount of STAT3 in the cytosol. Moreover, STX-0119 inhibited tumor growth in a subcutaneous model of lung cancer.

As shown in Fig. 2, STX-0119 exhibited a potent anti-proliferative and survival effect on lung cancer cells. To further investigate the apoptosis-inducing activity of STX-0119 in lung cancer cells, A549, H1299, and H23 cells were stained with an Annexin V/PI solution. We also observed the induction of PARP cleavage by STX-0119 in lung cancer cells (Fig. 3). Our results indicate that STX-0119 enhances apoptosis-inducing activity in lung cancer cells.

According to previous research, STX-0119 has a tumor-suppressive effect with no sign of side effects at a dose of 80 mg/kg [26, 31, 33]. To NOD-scid mice bearing glioblastoma cells, STX-0119 was orally administered at a dose of 80 mg/kg three times a week and a greater than 50% inhibition in tumor growth was shown [27]. There is also a report that STX-0119 reduces tumor growth by more than 50% in a subcutaneous model of lymphoma at a dose of 160 mg/kg (twice a week for the first week; once a week for the following 2 weeks) [28]. Based on those previous reports, we undertook to examine the effects of STX-0119 at doses of 80 mg/kg and 160 mg/kg on lung tumor growth in vivo. In our model, mice treated with 80 mg/kg STX-0119 showed no side effects, but the tumor growth suppressing effect was not significant from that in the control mice. However, the 160 mg/kg dose of STX-0119 had a marked effect on the suppression of tumor growth but also showed an increased level of immunosuppression (Table 1). In previous studies, mice are treated with STX-0119 for no longer than 3 weeks [27, 28]; therefore, we conducted an analysis of hematological and biochemical parameters of mice treated STX-0119 for 3 weeks. We observed that the 3-week treatment with STX-0119 resulted in a decreased WBC level, especially in eosinophils, neutrophils, lymphocytes, and monocytes. That result was expected as STAT3 activity is closely related to regulation of the immune system [34, 35]. Based on the observed immune system reaction, we conclude that STX-0119 can be a therapeutic agent, but there can be difficulties when it is used at a high dose.

To take advantage of the benefits of STX-0119 without inducing side effects, we suggest using STX-0119 as part of a combined therapy with present-day conventional lung cancer therapeutics, such as cisplatin. A low dose of STX-0119 may not produce a sufficient suppressive effect on the growth of tumor cells, but in the tumor microenvironment, it may work as a barrier for drug penetration [36]. There are previous hypotheses that targeting STAT3 will significantly reduce the immunosuppressive nature of the stroma, which is involved in STAT3-dependent cytokine networks [13, 35, 37]. Other research comparing the STX-0119 effects in nude and an immune-deficient NOG mouse with a deletion of both MHC-class I and class II genes demonstrated that STX-0119 can recover antitumor immune response patterns [29]. Another study indicated the importance of JAK-STAT3 in the formation of pre-metastatic niches, in which myeloid cells provide cytokines, growth factors, and other molecules, thereby allowing disseminated tumor cells to proliferate and resist apoptosis [38].

Developing combination therapies that would reduce side effects and improve efficacy has been a recent research trend. Cancer immunotherapies can offer a synergetic effect in treatment when used together, raising the possibility of improvements to clinical outcomes [39]. There is already a STAT3-related agent that has been approved by the FDA since 2006 for the treatment of renal cell carcinoma and imatinib-resistant gastrointestinal stromal tumor. Sunitinib, which blocks multiple tumor-associated tyrosine kinases, enhances antitumor effects by blocking immune system STAT3, decreasing the numbers and effectiveness of myeloid-derived suppressor and Treg cells [40] and affecting the tumor immunologic microenvironment. Other cancer-targeting agents are currently under investigation to determine their possible synergetic effects when used in combination with other treatments [39].

## Conclusions

In the present study, we examined the effect of STX-0119 on the viability of lung cancer cells as well as its possible biological mechanism, focusing on cell growth suppression and apoptosis. Also, by using an in vivo mouse model, we investigated the potency of STX-0119 as a therapeutic for NSCLC. However, a hematological side effect, which had not been previously reported, was observed. Consideration of the side effect suggests that STX-0119 would be better used in a combinational therapy; that is, adding a lower dose of STX-0119 to classic lung cancer therapeutics may be an effective strategy in cancer treatment. Further studies should be undertaken to examine the possible synergetic effects on tumor suppression, the tumor microenvironment, and associated biological mechanisms that could be provided by blocking STAT3 with STX-0119 in combination with the other chemotherapeutics.

## Data Availability

N/A
